# A bedside swallowing screen for the identification of post-extubation dysphagia on the intensive care unit – validation of the Gugging Swallowing Screen (GUSS)—ICU

**DOI:** 10.1186/s12871-023-02072-6

**Published:** 2023-04-13

**Authors:** Claudia Troll, Michaela Trapl-Grundschober, Yvonne Teuschl, Adrien Cerrito, Montserrat Gallego Compte, Martin Siegemund

**Affiliations:** 1 Department of Therapies, University Hospital Basel, Spitalstrasse 21, 4031 Basel, Switzerland; 2 University Hospital Tulln, Alter Ziegelweg 10, 3430 Tulln an Der Donau, Austria; 3 Department for Clinical Neurosciences and Preventive Medicine, University for Continuing Education Krems, Dr.-Karl-Dorrek-Strasse 30, 3500 Krems, Austria; 4 Bern University of Applied Sciences Health, Department of Health Professions, Murtenstrasse 10, 3008 Bern, Switzerland; 5 HNO Am Claraplatz, Claraplatz 1, 4058 Basel, Switzerland; 6 Intensive Care Unit, Department Acute Medicine, University Hospital Basel, Petersgraben 4, 4031 Basel, Switzerland

**Keywords:** Post-extubation dysphagia, Aspiration pneumonia, Gugging Swallowing Screen, Intensive Care Unit, Speech therapy

## Abstract

**Purpose:**

Screening for dysphagia at the intensive care unit (ICU) soon after extubation can prevent aspiration, pneumonia, lower mortality, and shorten re-feeding interval. This study aimed to modify the Gugging Swallowing Screen (GUSS), which was developed for acute stroke patients, and to validate it for extubated patients in the ICU.

**Methods:**

In this prospective study, forty-five patients who had been intubated for at least 24 h were recruited consecutively at the earliest 24 h after extubation. The modified GUSS-ICU was performed twice by two speech and language therapists independently. Concurrently, gold standard the flexible endoscopic evaluation of swallowing (FEES) was performed by an otorhinolaryngologist. Measurements were conducted within a three-hour period; all testers were blinded to each other’s results.

**Results:**

According to FEES, 36 of 45 (80%) participants were diagnosed with dysphagia; 13 of those were severe, 12 moderate, and 11 mild. Compared to FEES, the GUSS-ICU predicted dysphagia well (area under the curve for the initial rater pair: 0.923, 95% CI 0.832–1.000 and 0.923, 95% CI 0.836 -1.000 for the second rater pair). The sensitivity was 91.7% (95% CI 77.5–98.3%) and 94.4% (95% CI 81.3–99.3%); the specificity was 88.9% (51.8–99.7%) and 66.7% (29.9–92.5%); the positive predictive values were 97.1% (83.8–99.5%) and 91.9% (81.7–96.6%), and the negative predictive values were 72.7% (46.8–89%) and 75% (41.9–92.6%) for the first and second rater pairs, respectively.

Dysphagia severity classification according to FEES and GUSS-ICU correlated strongly (Spearman’s rho: 0.61 for rater 1 and 0.60 for rater 2, *p* < 0.001). Agreement by all testers was good (Krippendorffs Alpha: 0.73). The interrater reliability showed good agreement (Cohen`s Kappa: 0.84, *p* < 0.001).

**Conclusion:**

The GUSS-ICU is a simple, reliable, and valid multi-consistency bedside swallowing screen to identify post-extubation dysphagia at the ICU.

**Trial registration:**

ClinicalTrials.gov Identifier: NCT04532398,31/08/2020.

## Introduction

Aspiration pneumonia is the most serious consequence of dysphagia. It describes an infectious and inflammatory disease of the lungs caused by aspiration of secretions, liquids or food particles from the mouth or stomach contents. Acute dysphagia is common in intensive care medicine. It affects 29–47% of frail elderly admitted to the acute geriatric unit, up to 78% of acute stroke patients, and approximately 62% of critically ill patients who are intubated and mechanically ventilated [1–4].

Post-extubation dysphagia (PED) has been associated with a higher risk of pneumonia, increased duration of parenteral nutrition, reintubation, prolonged hospital and intensive care unit (ICU) stays, decreased quality of life, and an increased risk for death for up to 1 year after ICU admission [5–8]*.* Dysphagia increases healthcare utilization and costs, as has been shown across different clinical populations, particularly in stroke patients [9, 10]. Early detection and treatment of dysphagia may therefore prevent serious clinical complications such as aspiration pneumonia, improve patients’ outcome and save hospital resources [11]*.* While standardized protocols have been established to systematically assess dysphagia in acute stroke patients and are recommended by guidelines [12], no such protocols or guidelines have yet been published for the ICU [13]. However, experts recommend, in extubated critically ill patients on the ICU, a systematic bedside screening algorithm which include the water swallow test (WST) followed by expert comprehensive swallowing assessments of screening positive patients [14].

Instrumental examinations such as flexible endoscopic evaluation of swallowing (FEES) or videofluoroscopic swallowing study can be considered the gold standard. However, the equipment and the experts necessary for these techniques are not always available. A recent review searching for non-instrumental strategies that could serve for PED assessment in critically ill patients showed that swallowing assessments were primarily tested in mixed hospital populations or in stroke patients [14]. A combination of the WST with a Bedside Swallowing Evaluation (BSE) performed by speech and language therapists (SLTs) was the only strategy that has been validated for the identification of PED in acute respiratory failure survivors [15]*.* The Gugging Swallowing Screen (GUSS) might be suited for dysphagia screening at the ICU. It has been designed as a simple bedside screen that can be used by SLTs as well as by nurses and allows a graded assessment of the patient's swallowing abilities, and enables nutritional recommendations [16, 17]. The GUSS has previously been used for COVID-19 patients treated at the ICU [18] and in acute geriatric patients [2] but has only been validated for acute stroke patients [16]. In a previous attempt, the GUSS was partly adapted for the ICU population, but the GUSS-ICU has never been validated [19].

This study aimed to modify the GUSS for the ICU while retaining its important multi-consistency character and to test its validity. In this study, we tested the validity of this new multi-consistency GUSS-ICU compared to FESS and its interrater reliability for extubated patients in the ICU.

## Methods

### Study design

In this prospective, monocentric study in Switzerland, 45 patients who had been intubated at the ICU were recruited consecutively. The modified GUSS-ICU was performed twice, i.e. by two SLTs independently. The index test (FEES), was performed by an otorhinolaryngologist. All measurements were taken within three hours, and all testers were blinded to the test results of the others. The three assessments were performed in randomized order according to a computer-generated randomization list, and the information on treatment assignment was provided to the two SLTs by the principal investigator.

The GUSS-ICU study protocol was reviewed and approved by the Local Ethics Committee named Ethikkommission Nordwest-und Zentralschweiz EKNZ, approval number BASEC 2020-F01555. Written informed consent was obtained from all patients. Clinical Trial Registration: NCT 04,532,398,31/08/2020.

### Subjects

All patients who were intubated in the adult ICU of the university hospital Basel for at least 24 h between September 2020 and February 2021 were considered for recruitment. They had to be 18 years old or older, have a minimal mental status test score of 24, and extubation had to occur at least one hour prior to study participation.

### Modification of Gugging Swallowing Screen for the ICU

Because of the complex situation at the ICU, the heterogeneity of underlying diagnoses, and the variable degree of vigilance, items were added during the preliminary assessment phase of the GUSS to assure safety during swallowing.

**Fig. 1:**
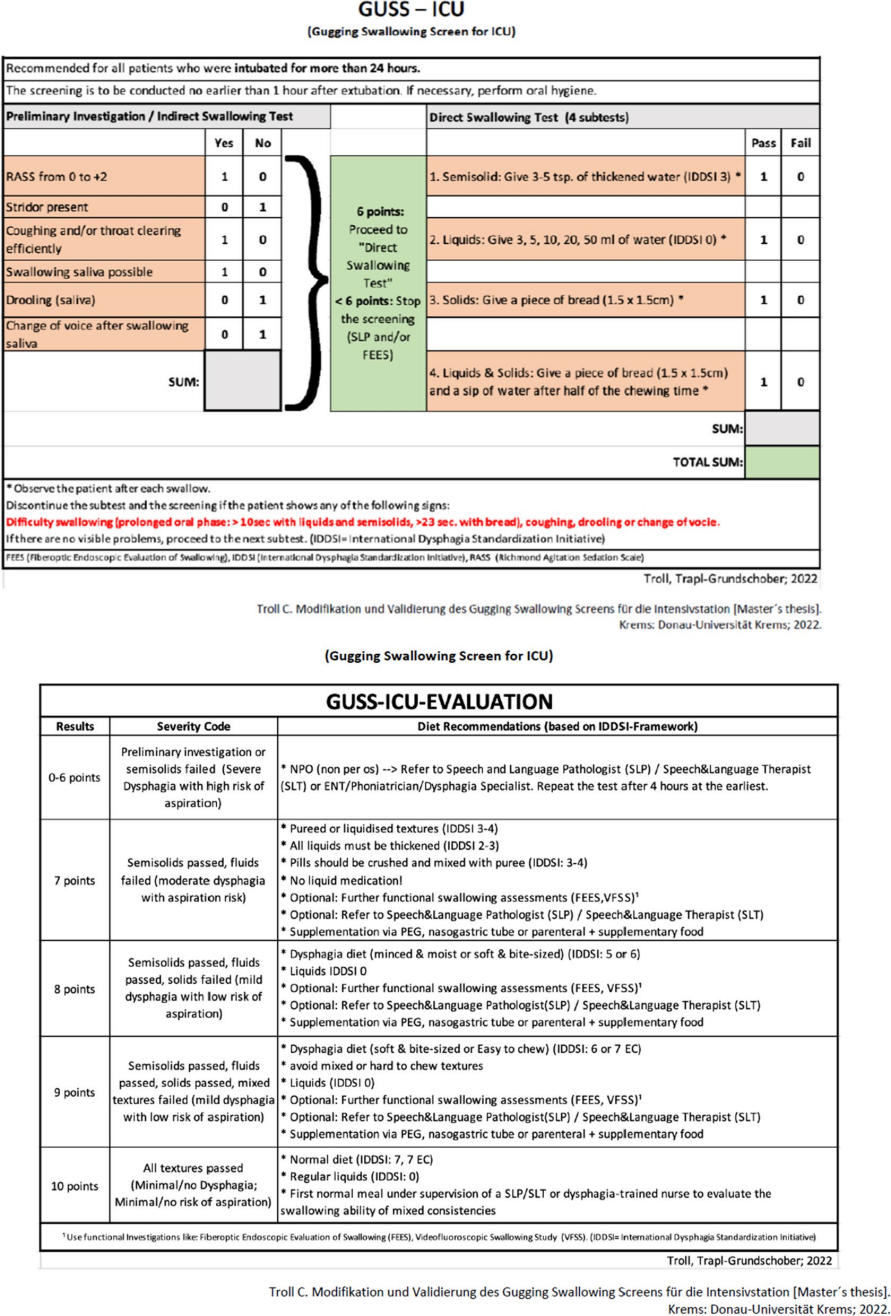
Assessment and Evaluation sheet, severity categories and dietary recommendations of the Gugging Swallowing Screen – Intensive Care Unit (GUSS-ICU)

Similar to the original GUSS, the GUSS-ICU consists of two parts, the indirect and the direct swallow test (Fig. 1). The first part, the indirect swallow test, consists of six items that do not necessarily have to be performed in order [16]. Compared to the original GUSS, the item “vigilance” was replaced by a Richmond Agitation Sedation Scale score of 0 to + 2 [20]*.* Furthermore, the presence of stridor is now assessed. Consistent with the original GUSS, the other items investigate whether coughing or throat clearing is effectively possible, the presence of drooling, or a voice change after swallowing saliva. One point is scored for each item when inconspicuous. If the maximum score of six points is not reached by the patient, the screening test must be stopped. That is, full completion of the first part is a prerequisite for the second part.

The second part, the direct swallow test, consists of four sequential subtests of fixed order. It starts with a diet level of International Dysphagia Diet Standardisation Initiative (IDDSI) 3 (moderately thick), followed by IDDSI 0 (thin), IDDSI 7 (solid), and finally with a mixed solid–liquid consistency. The prescribed sequence must be followed. The assessment criteria used in the direct swallowing test (four subtests) are: problems swallowing (prolonged oral phase: > ten seconds for thin liquid and moderately thick, > 23 s for solid food), coughing, drooling, or a change in voice. These criteria are tested in each subtest. A physiological swallow test scores one point, and a pathological one zero points. If a subtest scores zero, the examination must be terminated. Ten points are the highest score a patient can achieve in the GUSS-ICU and denotes normal swallowing function without risk of aspiration. Diet recommendations are given according to the score achieved in the GUSS-ICU (Fig. 1).

### FEES examination

The FEES was performed within 3 h of GUSS-ICU by an otorhinolaryngologist using a transnasal video endoscopy performed with a flexible rhino-laryngoscope. The swallow test by FEES was evaluated with saliva, then with different types of food consistencies (liquid, semisolid, solid), and finally with swallow portions of various solid sizes. The results of FEES were first graded according to the Penetration Aspiration Scale (PAS) of Rosenbek et al. [21]. The highest score achieved in either the semisolid or the fluid trial was taken as the final score. Swallowing dysfunction was then classified with a FEES based 4-grade dysphagia severity scale that has previously been developed and published. For the calculation of statistical results, the severity, as well as the general occurrence of dysphagia, was classified into the four severity levels [22] (Table 1).

**Table 1:** Comparison of dysphagia severity classification according to GUSS-ICU and FEES

**GUSS-ICU**		**FEES (based on Warnecke **[22]**)**	
0–6 Points	Severe dysphagia	3 Points	Severe dysphagia (penetration/aspiration events with two or more consistencies)
7 Points	Moderate dysphagia	2 Points	Moderate dysphagia (penetration/aspiration events with one consistency)
8–9 Points	Mild dysphagia	1 Points	Mild dysphagia (premature spillage and/or residues, but no penetration/aspiration events)
10 Points	No dysphagia	0 Points	No relevant dysphagia

### Statistical analysis

Concurrent validity was determined by comparing the GUSS-ICU to the reference standard FEES. The receiver operating characteristic curves (ROC) were plotted, and areas under the curves were calculated. For safety reasons, we chose to compare dysphagia versus no dysphagia. Dysphagia was defined for FEES as a grade of > 0 on the dysphagia severity scale by Warnecke to identify any pathological signs associated with dysphagia (primature spillage, penetration, aspiration) (Table 1, [22]). This corresponds to a PAS ≥ 1 in the Rosenbek scale [21]. For the GUSS-ICU, a score of less than < 10 points was chosen to identify dysphagia as this indicates any abnormality in screening.

Sensitivity, specificity, and positive and negative predictive values of the GUSS-ICU were calculated at these cut-offs. To determine the validity of dysphagia severity rating of the GUSS-ICU, the GUSS-ICU categories were correlated with the severity classification according to Warnecke using a Spearman rank correlation. Validity of the GUSS-ICU was determined for each of the two raters. In addition, a sensitivity analysis was performed by combining all test pairs into one data set and by calculating Krippendorff's alpha. The interrater reliability of the GUSS-ICU was calculated for dysphagia risk (GUSS-ICU 10) by calculating Cohen's kappa.

### Sample size calculation

The incidence of clinically relevant dysphagia was reported to be about 60% on the ICU [23]. Following the estimation for sensitivity analysis of Bujang & Adan 2016 [24], with an incidence of 60% and a power of 0.885, a minimum sample size of 32 patients is required. Ten percent were added to account for drop outs; this resulted in a sample size of 45 participants.

## Results

### Patient characteristics

**Fig. 2:**
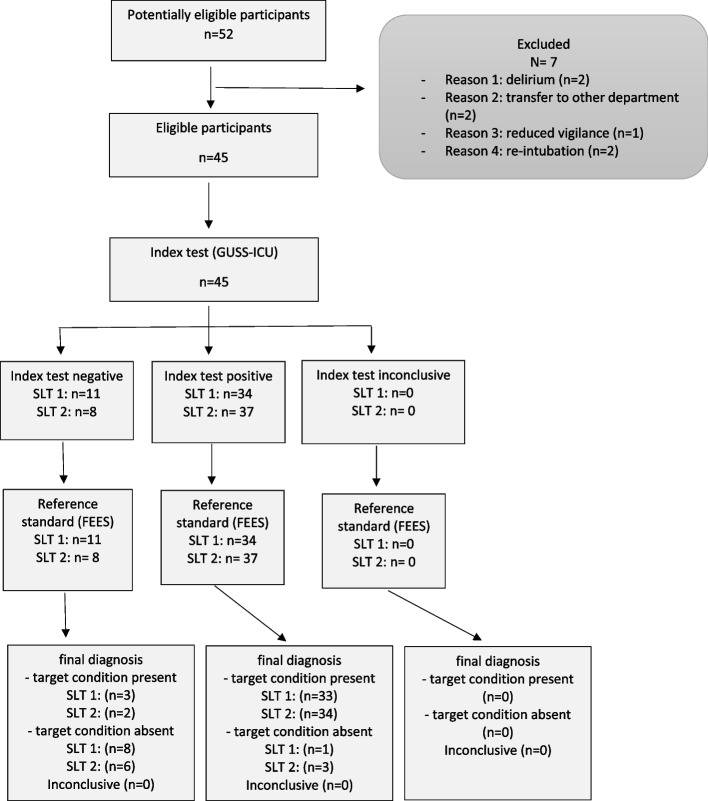
Flowchart of patients included in the study: Definition of abbreviations: SLT = speech and language therapist, FEES = flexible endoscopic evaluation of swallowing

Of the 52 patients fulfilling the inclusion criteria, 45 were included in the analysis (Fig. 2). Seven patients were excluded because of transfer to another department, delirium, reduced vigilance, re-intubation before the evaluation, or because reanimation became necessary. No patients had to be excluded because of missing or inconclusive data. No adverse events occurred during the index or the references test.The mean age was 63.3 years ± 11.7 standard deviation (SD); 47% were female.

The cause of intubation were medical and surgical cardiovascular disease (*n* = 19), acute pulmonary disease (*n* = 17), neurological disease (*n* = 3), sepsis (*n* = 3), multiorgan failure (*n* = 1), polytrauma (*n* = 1) and anaphylactic shock (*n* = 1).

The patients were intubated for an average of nine days and had an average Sequential Organ Failure Assessment (SOFA) score of 2.5 at extubation. The SOFA score is used to assess the degree of organ dysfunction and thereby determine the mortality risk [25]. Points are awarded from 0 (normal function) to 4 (massively restricted function).

According to FEES, 36 of 45 (80%) participants were diagnosed with dysphagia; 13 of those were severe, 12 moderate, and 11 mild.

The full study protocol can be obtained from the corresponding author.

### Validity

Using cut-off values of 0 points on the dysphagia severity scale by Warnecke for the FEES and 10 points for the GUSS-ICU, the comparisons yielded a sensitivity of 91.7% (95% CI 77.5–98.3%) and a specificity of 88.9% (51.8–99.7%) for the first rater pair and a sensitivity of 94.4% (81.3–99.3%), and a specificity of 66.7% (29.9–92.5%) for the second rater pair. The positive predicted values were 97.1% (83.8–99.5%) and 91.9% (81.7–96.6%), and the negative predictive values were 72.7% (46.8–89.0%) and 75.0% (41.9–92.6%), respectively, for rater 1 and rater 2 (Table 2). The positive likelihood ratios are 8.25 for the first and 2.83 for the second rater pair; the negative likelihood ratios are 0.09 for the first rater pair and 0.08 for the second pair.

**Table 2:** Sensitivity, specificity and predictives values of the GUSS-ICU for the diagnosis of dysphagia compared to flexible endoscopic evaluation of swallowing (FEES) for each of the two speech language therapists (SLT)

	**FEES** **Dysphagia positive**	**FEES** **Dysphagia negative**	
GUSS-ICU, SLT1 *n* = 45			
Dysphagia pos. < 10 points	33	1	PPV = 97.1% (95% CI 83.8–99.5%)
Dysphagia neg. = 10 points	3	8	NPV = 72.7% (95% CI 46.8–89.0%)
	Sensitivity = 91.7% (95% CI 77.5–98.3%)	Specifity = 88.9% (95% CI 51.8–99.7%)	Prevalence = 80% PLR = 8.25 (1.30–52.50), NLR = 0.09 (0.03–0.28)
GUSS-ICU, SLT2 *n* = 45			
Dysphagia pos. < 10 points	34	3	PPV = 91.9% (95% CI 81.7–96.6%)
Dysphagia neg. = 10 points	2	6	NPV = 75% (95% CI 41.9–92.6%)
	Sensitivity = 94.4% (95% CI 81.3–99.3%)	Specifity = 66.7% (95% CI 29.9–92.5%)	Prevalence = 80% PLR = 2,83, NLR = 0,08

NLR indicates negative likelihood ratio; *NPV* negative predictive value, *PLR* Positive Likelihood Ratio, *PPV* positive predictive value. Sensitivity, specifity, and predictive values of GUSS-ICU in the validation of ICU patients (*n* = 45) were compared with “gold standard” FEES results. Dysphagia at FEES was defined according to the dysphagia severity scale by Warnecke [22]

**Fig. 3:**
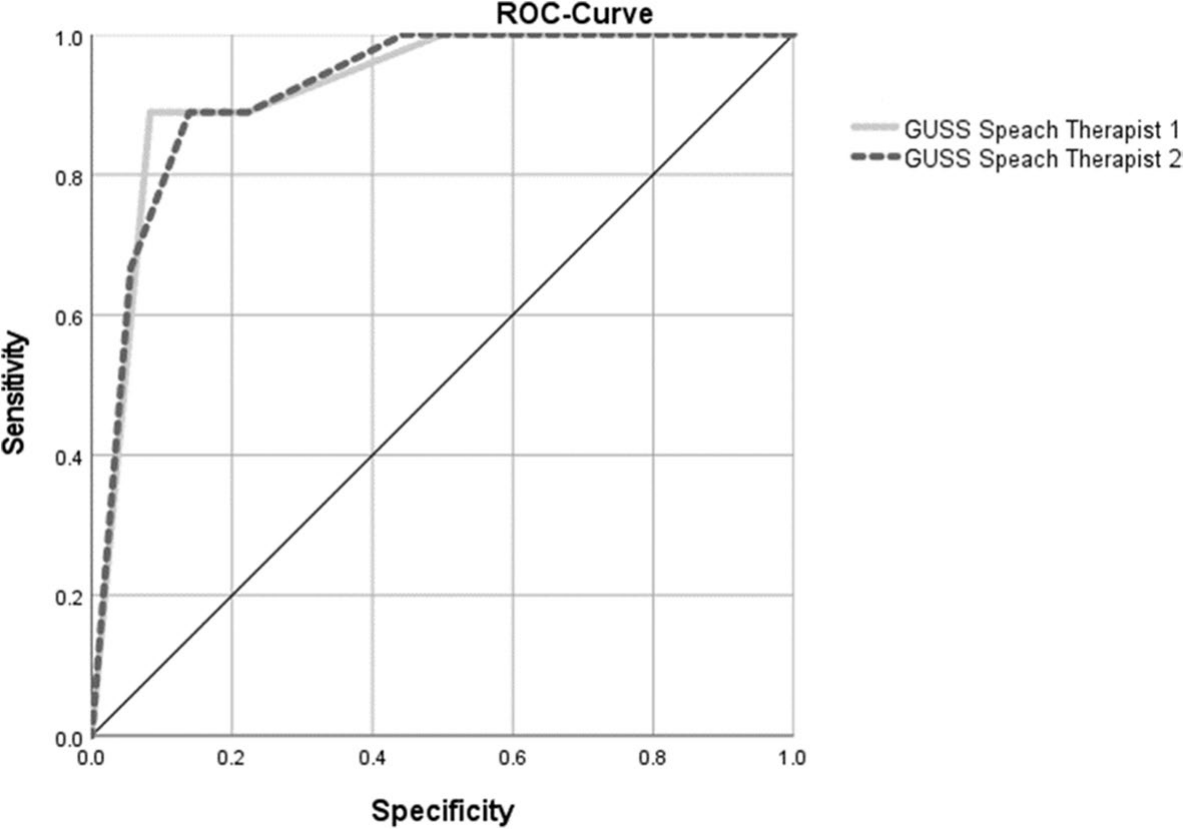
Receiver operating characteristic curve for both raters of the GUSS ICU as predictor for dysphagia as diagnosed by the FEES

The receiver operating characteristic curve (ROC) showed for both raters a good prediction of the aspiration risk by the GUSS-ICU (Fig. 3).

The area under the curve for the first raters was 0.923 (95% CI 0.832 to 1.000) and for the second raters, 0.923 (95% CI 0.836 to 1.000).The overall test accuracy between GUSS-ICU (rater 1) and FEES is 0.89, and between GUSS-ICU (rater 2) and FEES is 0.91.

The GUSS-ICU dysphagia severity classification showed a strong correlation with the FEES categories according to the dysphagia severity scale by Warnecke [22] (Spearman's rho for rater 1: 0.61, *p* < 0.001; for rater 2: 0.60, *p* < 0.001) (Table 3). The subsequent sensitivity analysis of all testers using Krippendorff`s alpha resulted in a coefficient of 0.73.

**Table 3:**
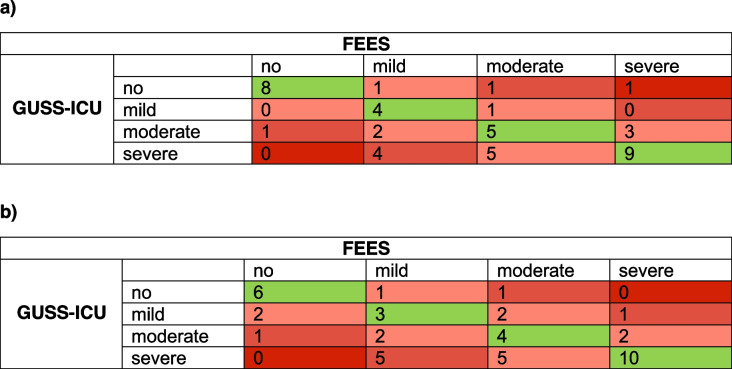
Comparison of the dysphagia severity classification according to FEES and to GUSS-ICU. Tables a and b represent the results of the two speech and language therapists, respectively

### Reliability

Dysphagia risk showed a strong agreement between the two rater (Cohen`s Kappa 0.84, *p* < 0.001).

## Discussion

In this study, we evaluated the validity and interrater reliability of the GUSS-ICU for identifying PED in the ICU. Compared to FEES, the GUSS-ICU showed 92 to 89% sensitivity, and 67% to 89% specificity in detecting patients with dysphagia, which is comparable to the original GUSS tested in stroke patients [16]. The interrater reliability between SLTs was good (Cohens kappa 0.84).

The major advantages of the GUSS-ICU are its simplicity and multi-consistency character, allowing provision of dietary recommendations according to the achieved score. In dysphagic patients, penetration and aspiration risk during swallowing varies according to the consistency of the bolus, with thin liquids having a higher risk compared to more viscous consistencies [26–28]. Multiconsistency screens such as the GUSS-ICU, in contrast to water testing [12] allow a stepwise approach for different liquid / food consistencies, thereby minimizing the risk of aspiration during testing. A retrospective database analysis of stroke patients showed that 22% of patients could benefit from multiconsistency screening compared to a water test because they received a special diet instead of non per os [29].

In this mixed-ICU population, 80% had dysphagia, according to FEES. As previously reported, the incidence of dysphagia is common in critical ill patients and higher when instrumental assessments are applied [3, 30]. However, a large number of mechanisms might be responsible for the development of dysphagia in the ICU [6]. Accordingly, dysphagia may resolve rapidly for some conditions or be persistent until hospital discharge in other patients and affect long-term outcome [7, 8, 18].

Thus, early use of dysphagia screening after extubation and regular reevaluations should be standard in an ICU.

Other dysphagia screenings have been suggested for the ICU, however the GUSS-ICU is currently the only validated multi-consistency screening. The Nurse-performed Screening (NPS) for example has only been analysed retrospectively and has not been validated compared to an instrumental swallowing assessment [31]. The Yale Swallow Protocol may not be performed on patients with a tracheal cannula or a nasogastric tube in place which limits its applicablility in ICU Patients [32]. The GUSS-ICU was performed without complications on two patients with a tracheal cannula. The only precondition in tracheostomized patients is, that the cuff should be deflated for the screening and a speaking valve is in place. The Volume-Viscosity Swallowing Test (V-VVST), a multiconsistency test, has not been validated for the ICU and might only be suited for SLT because of its complexity [33]. Furthermore, the diet recommendations are not classified according to the International Dysphagia Diet Standardisation Initiative (IDDSI), which makes a transfer to international diet management systems difficult. Recently, a bedside evaluation of swallowing function showed good accuracy to detect PED in critically ill patients after extubation [34]. However, this screening has been validated on physiotherapists and does not evaluate bolus swallows.

An international survey reported, that only 4% of dysphagia specialists are dedicated to an ICU, and only 66% of ICUs have the possibility of a SLT consultation. In addition, 77% of these wards reported not using standardized dysphagia screenings [13]. Therefore an easy-to-use, standardized bedside dysphagia screening tool, such as the GUSS-ICU, would allow ICU nurses to perform a systematic testing for dysphagia in the absence of dysphagia experts and/or instrumental swallowing evaluations. A recent study showed, that the FEES was not performed in 56% of critically ill patients after extubation because of the unavailability of FEES operators [34]. So far, the GUSS-ICU has not been validated for nurses in the ICU, but the original GUSS has been validated and successfully used by nurses in stroke patients [16, 35]. In a next step we plan to validate the GUSS-ICU for nurses. The GUSS-ICU is not intended to replace, but to complement, instrumental swallowing diagnostics or a clinical swallowing examination by a speech therapist. Bedside screenings performed by nurses, should be part of a dysphagia algorithm in the ICU in the future to be able to detect early risk of dysphagia and reduce the pulmonary complications after extubation. Even though the present validation study was performed exclusively on patients intubated for at least 24 h, all patients should be screened in the ICU.

Limitations of this study are its monocentric design and the relatively small sample size which is however comparable to other validation studies in this field. Furthermore, the heterogeneity of the underlying disease, the fluctuation in vigilance and the changes in medication at the ICU increase variability and lower generalizability. Repeated assessments with the GUSS-ICU and recording of vigilance at the time of testing may increase the accuracy of the screening.

## Conclusion

The GUSS-ICU is a simple, reliable, sensitive and valid multi-consistency bedside swallowing screen to identify PED at the ICU and should be used in the future by nurses on the ICU to screen systematically for dysphagia.

## Data Availability

Data are available from the corresponding author on reasonable request.

## References

[1] Baijens LW, Clavé P, Cras P (2016). European Society for Swallowing Disorders - European Union Geriatric Medicine Society white paper: oropharyngeal dysphagia as a geriatric syndrome. Clin Interv Aging.

[2] Poulsen SH, Rosenvinge PM, Modlinski RM (2021). Signs of dysphagia and associated outcomes regarding mortality, length of hospital stay and readmissions in acute geriatric patients: Observational prospective study. Clin Nutr ESPEN.

[3] Martino R, Foley N, Bhogal S (2005). Dysphagia after stroke: incidence, diagnosis, and pulmonary complications. Stroke.

[4] McIntyre M, Chimunda T, Koppa M (2022). Risk Factors for Postextubation Dysphagia: A Systematic Review and Meta-analysis. Laryngoscope.

[5] Macht M, Wimbish T, Clark BJ (2011). Postextubation dysphagia is persistent and associated with poor outcomes in survivors of critical illness. Crit Care Lond Engl.

[6] Macht M, Wimbish T, Bodine C, Moss M (2013). ICU-acquired swallowing disorders. Crit Care Med.

[7] Schefold JC, Berger D, Zürcher P (2017). Dysphagia in Mechanically Ventilated ICU Patients (DYnAMICS): A Prospective Observational Trial. Crit Care Med.

[8] Zuercher P, Moser M, Waskowski J (2022). Dysphagia Post-Extubation Affects Long-Term Mortality in Mixed Adult ICU Patients-Data From a Large Prospective Observational Study With Systematic Dysphagia Screening. Crit Care Explor.

[9] Attrill S, White S, Murray J (2018). Impact of oropharyngeal dysphagia on healthcare cost and length of stay in hospital: a systematic review. BMC Health Serv Res.

[10] Marin S, Serra-Prat M, Ortega O, Clavé P (2020). Healthcare-related cost of oropharyngeal dysphagia and its complications pneumonia and malnutrition after stroke: a systematic review. BMJ Open.

[11] Sherman V, Greco E, Martino R (2021). The Benefit of Dysphagia Screening in Adult Patients With Stroke: A Meta-Analysis. J Am Heart Assoc.

[12] Dziewas R, Michou E, Trapl-Grundschober M (2021). European Stroke Organisation and European Society for Swallowing Disorders guideline for the diagnosis and treatment of post-stroke dysphagia. Eur Stroke.

[13] Spronk PE, Spronk LEJ, Egerod I (2022). Dysphagia in Intensive Care Evaluation (DICE): An International Cross-Sectional Survey. Dysphagia.

[14] Perren A, Zürcher P, Schefold JC (2019). Clinical Approaches to Assess Post-extubation Dysphagia (PED) in the Critically Ill. Dysphagia.

[15] Lynch YT, Clark BJ, Macht M (2017). The accuracy of the bedside swallowing evaluation for detecting aspiration in survivors of acute respiratory failure. J Crit Care.

[16] Trapl M, Enderle P, Nowotny M (2007). Dysphagia bedside screening for acute-stroke patients: the Gugging Swallowing Screen. Stroke.

[17] Warnecke T, Im S, Kaiser C (2017). Aspiration and dysphagia screening in acute stroke - the Gugging Swallowing Screen revisited. Eur J Neurol.

[18] Ceruti S, Glotta A, Galli A (2012). (2021) Dysphagic disorder in a cohort of COVID-19 patients: Evaluation and evolution. Ann Med Surg.

[19] Christensen M, Trapl M (2018). Development of a modified swallowing screening tool to manage post-extubation dysphagia. Nurs Crit Care.

[20] Ely EW, Truman B, Shintani A (2003). Monitoring sedation status over time in ICU patients: reliability and validity of the Richmond Agitation-Sedation Scale (RASS). JAMA.

[21] Rosenbek JC, Robbins JA, Roecker EB (1996). A penetration-aspiration scale. Dysphagia.

[22] Warnecke T, Dziewas R, Martin CR, Preedy VR (2014). Chapter 106—swallowing in progressive supranuclear palsy and implications for nutrition. Diet and Nutrition in Dementia and Cognitive Decline.

[23] Morgan AS, Mackay LE (1999). Causes and complications associated with swallowing disorders in traumatic brain injury. J Head Trauma Rehabil.

[24] Bujang MA, Adnan TH (2016). Requirements for Minimum Sample Size for Sensitivity and Specificity Analysis. J Clin Diagn Res.

[25] Vincent JL, Moreno R, Takala J (1996). The SOFA (Sepsis-related Organ Failure Assessment) score to describe organ dysfunction/failure. On behalf of the Working Group on Sepsis-Related Problems of the European Society of Intensive Care Medicine. Intensive Care Med.

[26] Clavé P, de Kraa M, Arreola V (2006). The effect of bolus viscosity on swallowing function in neurogenic dysphagia. Aliment Pharmacol Ther.

[27] Francesco M, Nicole P, Letizia S (2022). Mixed Consistencies in Dysphagic Patients: A Myth to Dispel. Dysphagia.

[28] Marques CHD, de Rosso ALZ, André C (2008). Bedside assessment of swallowing in stroke: water tests are not enough. Top Stroke Rehabil.

[29] Teuschl Y, Trapl M, Ratajczak P (2018). Systematic dysphagia screening and dietary modifications to reduce stroke-associated pneumonia rates in a stroke-unit. PloS One.

[30] McIntyre M, Doeltgen S, Dalton N (2021). Post-extubation dysphagia incidence in critically ill patients: A systematic review and meta-analysis. Aust Crit Care Off J Confed Aust Crit Care Nurses.

[31] See KC, Peng SY, Phua J (2016). Nurse-performed screening for postextubation dysphagia: a retrospective cohort study in critically ill medical patients. Crit Care Lond Engl.

[32] Suiter DM, Sloggy J, Leder SB (2014). Validation of the Yale Swallow Protocol: a prospective double-blinded videofluoroscopic study. Dysphagia.

[33] Clavé P, Arreola V, Romea M (2008). Accuracy of the volume-viscosity swallow test for clinical screening of oropharyngeal dysphagia and aspiration. Clin Nutr Edinb Scotl.

[34] Maamar A, Parent V, Prudhomme E, Guérot E, Berneau P, Frérou A, Le Tulzo Y, Jégoux F, Gacouin A, Tadié JM (2022). Fiberoptic endoscopic validation of a clinical screening test of swallowing function in critically ill patients performed within 24 h after extubation. J Crit Care..

[35] Palli C, Fandler S, Doppelhofer K (2017). Early Dysphagia Screening by Trained Nurses Reduces Pneumonia Rate in Stroke Patients: A Clinical Intervention Study. Stroke.

